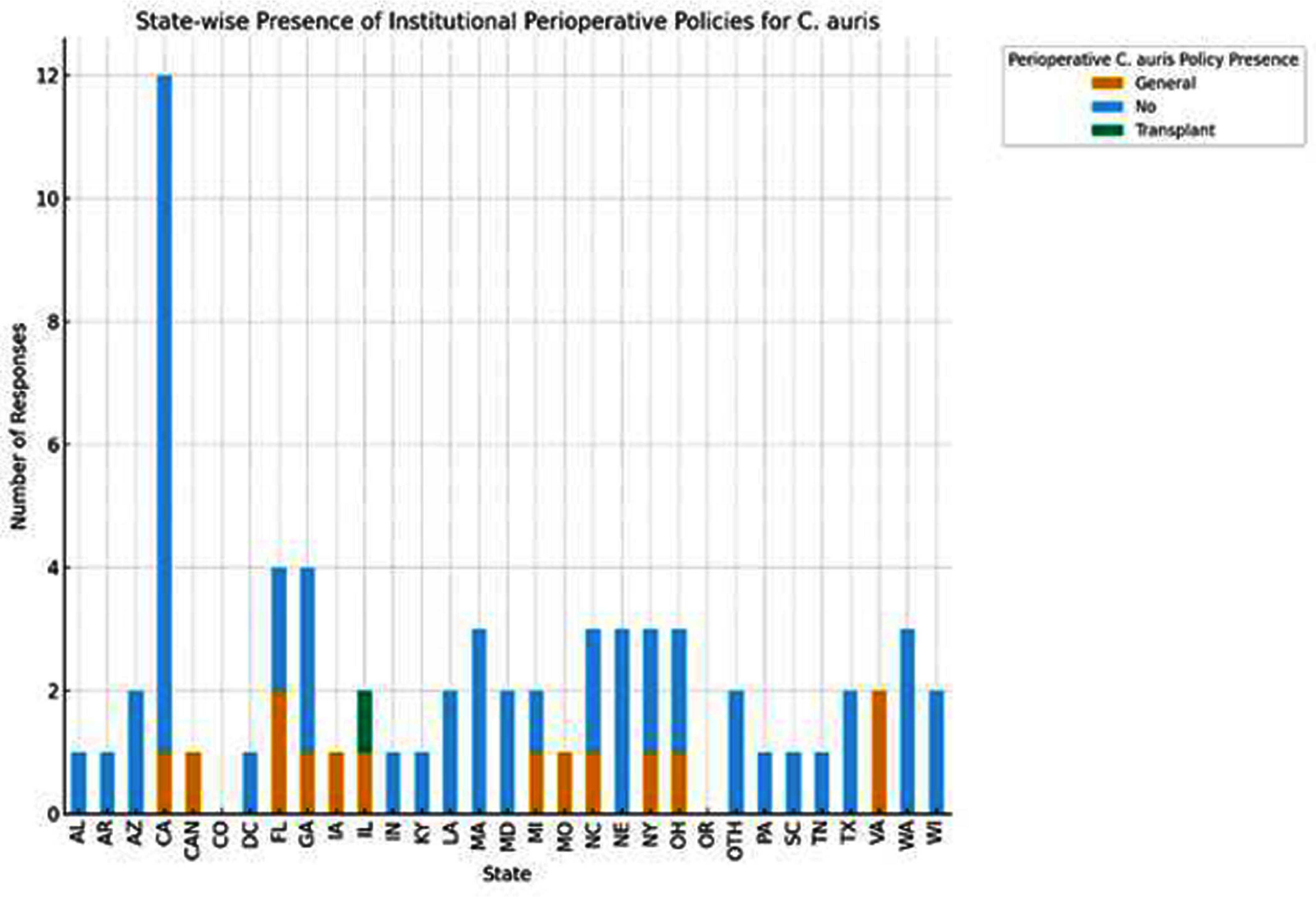# Reported Perioperative Practices for Candida auris Colonization among Surgical and Transplant Patients

**DOI:** 10.1017/ash.2025.333

**Published:** 2025-09-24

**Authors:** Dana Hassneiah, Matthew Lee, Preeti Mehrotra, Dana Pepe, Philip Polgreen, Susan E. Beekmann

**Affiliations:** 1 Beth Israel Deaconess Medical Center; 2 Bidmc; 3 University of Iowa Hospitals and Clinics; 4 University of Iowa

## Abstract

**Background:** Candida auris is an emerging multidrug-resistant pathogen that presents significant infection control challenges due to its ability to cause invasive infections and outbreaks. Despite its increasing prevalence in healthcare settings, there is limited guidance or data related to perioperative infection prevention in patients colonized with C.auris.(1-4) CDC guidelines do not address perioperative screening or management of C.auris in surgical patients, including solid organ transplant recipients.(5)

This study examines the reported experiences and approaches infectious diseases professionals take in managing C.auris colonization perioperatively. **Methods:** An online “Quick Query” poll was distributed to members of the Emerging Infections Network (EIN). EIN Quick Queries are a unique tool that utilize focused rapidly-deployable polls to ascertain members’ opinions/approaches to emerging infectious diseases with limited data, allowing for hypothesis generation or identifying areas of need. Our poll included 6 questions assessing perioperative prevention of C.auris infection. Descriptive analysis was used to evaluate responses. **Results:** Eighty-four EIN members completed the poll, representing 29 U.S. states and three other countries/territories. California accounted for the largest share of responses (17%), followed by Massachusetts (7%), and Florida (6%). Institutional protocols for C.auris prevention/management in surgical patients existed in 17% (14/84) of facilities, with only one facility reporting transplant-specific guidelines (Figure 1). Preoperative screening for C. auris was reported by 8% (7/84) of respondents, predominantly from California (5/7). Among the 44 respondents who encountered colonized surgical patients, only four (9%) used perioperative antifungal prophylaxis, with micafungin as the preferred agent. Notably, none of the seven sites utilizing preoperative screening reported using perioperative anti-fungal prophylaxis for C. auris colonization. Three of the four respondents who used perioperative antifungal prophylaxis also reported encountering postoperative C.auris infections in colonized surgical patients, suggesting evolving practices influenced by clinical outcomes. C.auris colonization in transplant recipients prior to transplantation was reported by 11 respondents (13%), while only two (2%) reported encounters involving transplants from colonized donors. In total,19% of respondents (16/84) reported postoperative invasive C.auris infections in previously colonized patients, including 13 surgical patients and 3 transplant recipients. Three other cases were described among transplant recipients without known donor or recipient colonization pre-transplant. **Conclusion:** The survey highlights the lack of institutional guidelines for Candida auris prevention in perioperative settings, including among solid-organ transplant patients. Postoperative C.auris infections are being encountered, although uncommon, underscoring the need for further research and standardized guidelines to address perioperative prevention of this emerging pathogen.

**Figure:**